# Clinical Follow-Up and Postmortem Findings in a Cat That Was Cured of Feline Infectious Peritonitis with an Oral Antiviral Drug Containing GS-441524

**DOI:** 10.3390/v14092040

**Published:** 2022-09-14

**Authors:** Daniela Krentz, Katharina Zwicklbauer, Sandra Felten, Michèle Bergmann, Roswitha Dorsch, Regina Hofmann-Lehmann, Marina L. Meli, Andrea M. Spiri, Ulrich von Both, Martin Alberer, Anne Hönl, Kaspar Matiasek, Katrin Hartmann

**Affiliations:** 1 Clinic of Small Animal Medicine, Centre for Clinical Veterinary Medicine, LMU Munich, D-80539 Munich, Germany; 2 Clinical Laboratory, Department of Clinical Diagnostics and Services, Center for Clinical Studies, Vetsuisse Faculty, University of Zurich, CH-8057 Zurich, Switzerland; 3 Division of Paediatric Infectious Diseases, Dr. von Hauner Children’s Hospital, University Hospital, LMU Munich, D-80337 Munich, Germany; 4 Section of Clinical and Comparative Neuropathology, Institute of Veterinary Pathology, Centre for Clinical Veterinary Medicine, LMU Munich, D-80539 Munich, Germany

**Keywords:** FCoV, FIP, Mutian, Xraphconn^®^, antiviral chemotherapy, feline coronavirus, therapy, treatment, necropsy

## Abstract

This is the first report on a clinical follow-up and postmortem examination of a cat that had been cured of feline infectious peritonitis (FIP) with ocular manifestation by successful treatment with an oral multicomponent drug containing GS-441524. The cat was 6 months old when clinical signs (recurrent fever, lethargy, lack of appetite, and fulminant anterior uveitis) appeared. FIP was diagnosed by ocular tissue immunohistochemistry after enucleation of the affected eye. The cat was a participant in a FIP treatment study, which was published recently. However, 240 days after leaving the clinic healthy, and 164 days after the end of the 84 days of treatment, the cured cat died in a road traffic accident. Upon full postmortem examination, including histopathology and immunohistochemistry, there were no residual FIP lesions observed apart from a generalized lymphadenopathy due to massive lymphoid hyperplasia. Neither feline coronavirus (FCoV) RNA nor FCoV antigen were identified by quantitative reverse transcription polymerase chain reaction (RT-qPCR) and immunohistochemistry, respectively, in any tissues or body fluids, including feces. These results prove that oral treatment with GS-441524 leads to the cure of FIP-associated changes and the elimination of FCoV from all tissues.

## 1. Introduction

Feline infectious peritonitis (FIP) caused by the feline coronavirus (FCoV) is an infectious disease that occurs in felids worldwide. Infection with wildtype FCoV initially only causes a harmless intestinal infection. Mutation of the virus within the host, however, can lead to the disease FIP, which once clinically apparent is always fatal within a short period of time if left untreated [1]. Hitherto identified mutations mainly result in changes in coronaviral spike proteins, which enable the virus to replicate effectively within macrophages and to spread within the cat [2,3,4]. Subsequent activation of the immune system leads to extensive cytokine release, and thereby exaggerated multisystemic inflammatory lesions. The average survival time without effective treatment is only 8 days after diagnosis [1], and most cats have to be euthanized early due to their severe condition. However, recent studies have demonstrated the efficacy of antiviral compounds containing the nucleoside analog GS-441524 in cats with FIP [5,6,7]. Although successful clinical recovery from FIP has previously been reported [7], this case report is the first description of the complete recovery in a cat whose tissues could be examined after a fatal road traffic accident via necropsy, including histopathology as well as FCoV immunohistochemistry (IHC) and quantitative reverse transcription polymerase chain reaction (RT-qPCR).

In the first controlled study (performed by the same study group) using an oral multicomponent compound called Xraphconn^®^, provided by Mutian Life Sciences Limited, containing GS-441524, 18 cats with naturally occurring and confirmed FIP were treated daily over 84 days [7]. All 18 cats recovered with dramatic resolution of all clinical and laboratory parameters, disappearance of effusion, and complete improvement of neurological signs, if present. Quantitative assessment revealed a large reduction in viral loads (across all measured compartments) within the first few days of treatment. Treatment with Xraphconn^®^ containing GS-441524 was highly effective against FIP, without causing clinically relevant adverse effects [7,8].

One cat participating in the abovementioned study [7] died in an unobserved road traffic accident 164 days after the end of treatment. The aim of the present case report was (1) to describe the clinical course during and after treatment, (2) to screen for pathological sequelae of FIP in a cat treated with an oral antiviral drug by examining tissue samples via necropsy and histopathology, and (3) to search for FCoV antigen and viral RNA by IHC and RT-qPCR, respectively.

## 2. Case Description

### 2.1. Signalment and History

A male, neutered, 6-month-old European Shorthair cat was initially presented to a local veterinarian in February 2021. Three of the eight litter mates had died of FIP. One of the siblings that also suffered from confirmed FIP (ocular and neurological manifestation) was another study participant, and was also cured [7]. According to the owner, the cat developed clinical signs of recurrent fever, lethargy, and lack of appetite at the end of January 2021. The cat tested negative for feline immunodeficiency virus (FIV) and feline leukemia virus (FeLV). On admission to the local veterinarian at the beginning of February 2021, the cat showed signs of anisocoria, with a relatively miotic, round, and poorly responsive pupil in the left eye (OS). The OS further revealed rubeosis iridis, with marked thickening and bulging of the iris and fibrin precipitates within the anterior eye segment. The intraocular pressure at this time was within normal limits (OS, 11 mmHg; right eye (OD), 16 mmHg). Blood work at the initial examination revealed regenerative anemia (hematocrit, 21.2%; reference range (RR), 29.7–44.5%) and a reticulocyte count of 50.2 × 10^9^/L; all other hematology parameters were unremarkable. The altered hematologic parameters were consistent with FIP. However, the cat did not show signs of neutrophilia or lymphopenia, which can occur in cats with FIP [9]. In the follow-up examination (10 days later), the left globe appeared larger than the right globe. Fibrin deposition within the anterior segment had increased, and was accompanied by a severe hyphema. Fundoscopy revealed partial retinal detachment. The intraocular pressure had increased to 23 mmHg OS (OD 13 mmHg). The OS was enucleated after a third examination 5 days later, due to continuous deterioration and evidence of high-grade anterior uveitis with an intraocular pressure of 48 mmHg (OD 16 mmHg).

The enucleated OS was subjected to histopathological examination. Marked pyogranulomatous uveitis and optic neuritis with retinal detachment were identified. FCoV IHC (as described in 2.5) revealed multiple intralesional immunostaining-positive macrophages, confirming, in combination with the ophthalmologic examination, an ocular manifestation of FIP. Additionally, FIP was confirmed by a positive RT-qPCR result of the ocular tissue (as described in 2.6 and shown in Figure 7).

### 2.2. Improvement of Clinical Signs, Ultrasonographic Findings, and Laboratory Abnormalities during Treatment

Before starting treatment (day 0), a complete physical examination, including determination of the Karnofsky score (see Table 1) [10,11], as well as hematology, serum biochemistry, and a detailed abdominal ultrasound, was performed. The Karnofsky score modified for cats was used to evaluate the general condition and well-being of the cats. The score ranges from 0% (dead), to 100%, which corresponds to a cat with healthy normal general condition [10,11].

**Figure 1:**
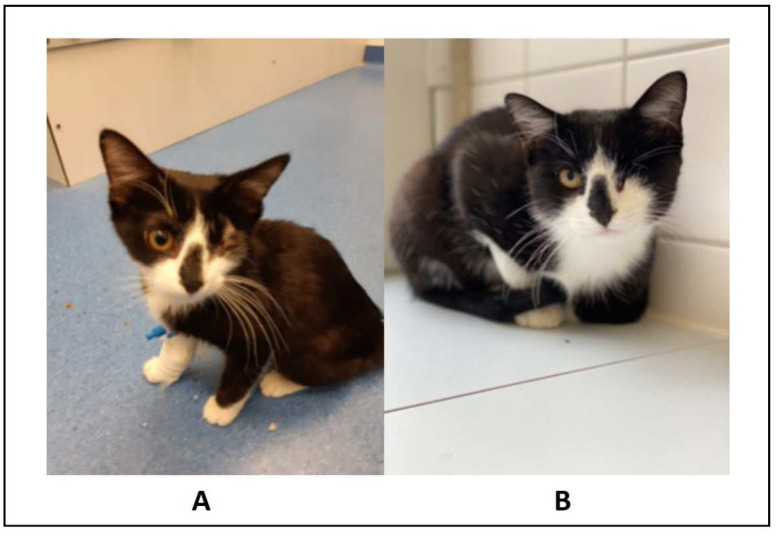
Pictures of the cat (**A**) on day 0 (day of first presentation in the clinic) and (**B**) on day 168, 12 weeks after the end of treatment.

In the clinic, the cat presented with reduced general condition, pale mucous membranes, dehydration, a body temperature of 39.1 °C, and a body condition score of 3/9 (Figure 1A). The cat had a Karnofsky score of 70% on day 0. At presentation, and the start of the study, the cat had a body weight of 1.8 kg, measured using a baby scale (AE Adam MTB 20 baby scale, Felde, Germany). Physical examination and determination of the Karnofsky score were performed daily during hospitalization in the clinic (day 0 to day 7) and at all rechecks on days 14, 28, 56, 83, and 168.

The cat was orally treated by daily administration of the multicomponent drug Xraphconn^®^ (Mutian Life Sciences Limited, Nantong, China) containing the nucleoside analog GS-441524, for 84 days. Due to the ocular manifestation, a dose of supposedly 10 mg/kg (according to the manufacturer) was chosen, with the drug being administered according to manufacturer’s instructions [7].

During hospitalization, the cat received supportive fluid therapy comprising Ringer’s lactate with potassium supplementation at 20 mval/L to control dehydration at individual dosage, calculated by rehydration and maintenance needs. On the first day of treatment (day 0), the cat developed a fever (40.5 °C), whereupon it received a single injection of metamizole (30 mg/kg) intravenously (IV).

**Figure 2:**
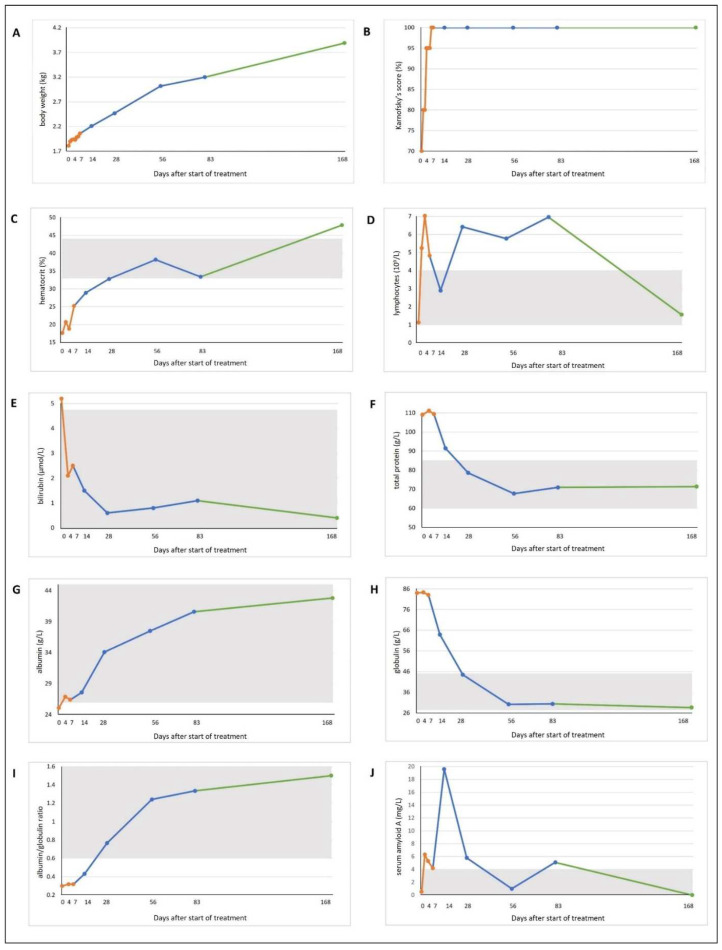
Timeline visualizing improvement of clinical and laboratory parameters throughout the study course. Lines are colored according to the different study sections: orange, hospitalization (day 0–7); blue, recheck visits during treatment (day 14, 28, 56, 83); green, follow-up period at the end of treatment. Grey shading marks the reference ranges, if present. (**A**) Body weight. (**B**) Karnofsky score modified for cats [10]. (**C**) Hematocrit. (**D**) Lymphocyte count. (**E**) Bilirubin concentration. (**F**) Total protein concentration. (**G**) Albumin concentration. (**H**) Globulin concentration. (**I**) Albumin/globulin ratio. (**J**) Serum amyloid A concentration.

From the second day of treatment onward, the cat’s appetite improved, and it started to gain weight. The cat was discharged from the clinic on day 7 with a body weight of 2.1 kg. At home, the weight continued to increase steadily, and the cat doubled the initial weight on day 56. Twelve weeks after the end of treatment (day 168), the cat had reached a weight of 4.0 kg (Figure 1B and Figure 2A and Table 2). The Karnofsky score increased to 80% on day 1, and reached 100% on day 7 (Figure 2B). Body temperature decreased to 38.5 °C on day 1, and remained normal for the rest of the study period (Table 2).

**Figure 3:**
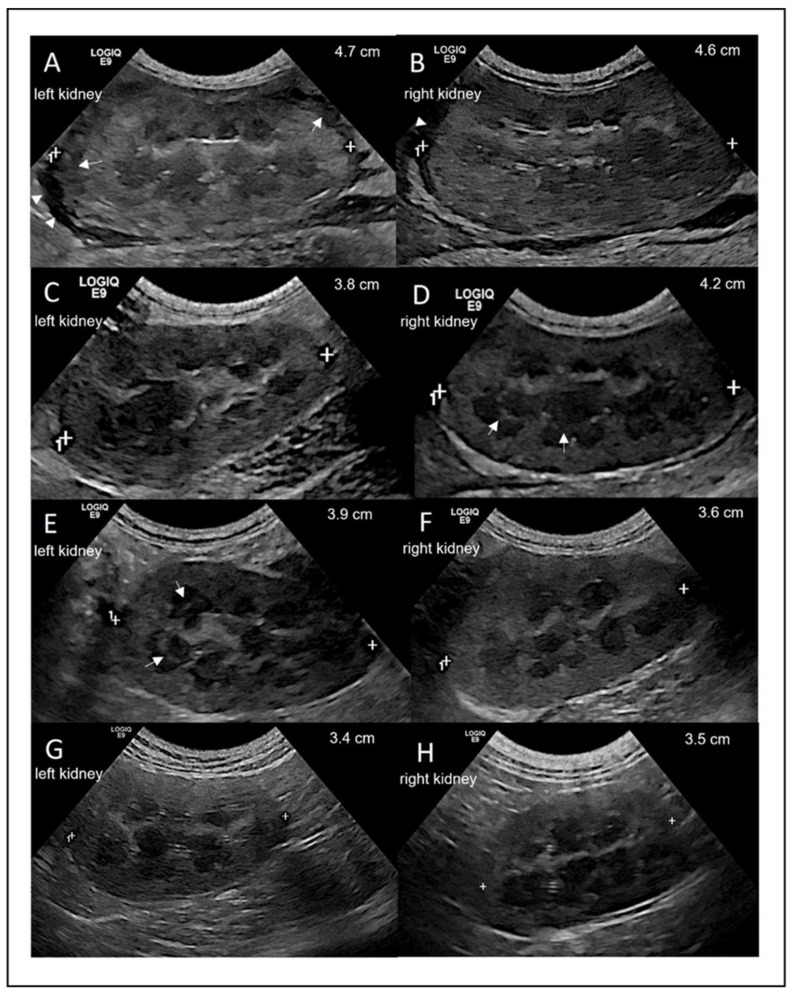
Ultrasonographic longitudinal view of the left and right kidney on days 0, 7, 14, and 168. (**A**,**B**) Day 0 with arrow heads showing free fluid in the retroperitoneal area and arrows showing hypoechoic subcapsular rim. (**C**,**D**) Day 7 with arrows showing the medullary rim sign. (**E**,**F**) Day 14 with arrows showing the medullary rim sign. (**G**,**H**) Day 168 with normal size, structure, texture, and echogenicity.

Abdominal ultrasound was performed using the Logiq E9 ultrasound machine (GE Healthcare) and an 8-MHz microconvex probe, with the cat in dorsal recumbency after clipping the fur. Upon presentation (day 0), the most notable finding was bilateral renomegaly. Regarding longitudinal measurements, the left and right kidney were 4.7 cm and 4.6 cm in size, respectively (Figure 3A,B), with a hypoechoic subcapsular rim on both sides. The surface of the left kidney was irregular, and the cortical parenchyma of both kidneys appeared hyperechoic and mottled. There was poor corticomedullary definition and a small amount of anechoic fluid in the retroperitoneal space. Intestinal lymph nodes were mildly enlarged with a homogeneous texture.

On day 7, the lengths of the left and right kidneys had decreased to 3.8 and 4.2 cm, respectively (Figure 3C,D). The cortex of both kidneys had a homogenous texture, and both kidneys had a distinct corticomedullary definition. The retroperitoneal fluid was no longer visible. A poorly defined medullary rim sign was observed in the right kidney on day 7 (Figure 3D) and in the left kidney on day 14 (Figure 3E). Both kidneys were considered normal in size, structure, texture, and echogenicity on day 56. The intestinal lymph nodes remained mildly enlarged throughout the study period.

Hematology was performed on days 0, 2, 4, 7, 14, 28, 56, 83, and 168 using an automatic analyzer (Cell-Dyn 3500, Abott Laboratories, Chicago, IL, USA). Differential blood count was additionally performed manually on blood smears exposed to Haema Quick Staining/Diff-Quick staining (LT-SYS^®^, Eberhard Lehmann GmbH, Berlin, Germany) if hematology parameters were abnormal. On day 0, the cat presented with severe non-regenerative, hypochromic, and microcytic anemia, and moderate thrombocytopenia. On day 2, hematocrit and reticulocyte counts increased, indicating early regeneration. On day 7, the cat was discharged from the clinic with a hematocrit of 0.252 L/L. On day 56, anemia had resolved (Table 2 and Figure 2C). By day 2, the lymphocytes, which were initially within the lower RR (day 0), had transitioned into mild lymphocytosis. Thrombocyte count was within the RR. Throughout the rest of the treatment period, lymphocyte counts indicated mild lymphocytosis, but 12 weeks after the end of treatment, the lymphocyte count was within the RR (Table 2 and Figure 2D). All other hematology parameters were within the RR throughout the entire study period (Table 2).

Serum biochemistry parameters were measured on days 0, 4, 7, 14, 28, 56, 83, and 168 using an automatic analyzer (Hitachi 911, Roche, Grenzach-Wyhlen, Germany). Symmetric dimethylarginine (SDMA) concentration was analyzed at IDEXX Diavet AG (Bäch, Switzerland) using a high-throughput immunoassay, and serum amyloid A (SAA) concentration was determined using a latex agglutination turbidimetric immunoassay reaction (LZ Test SAA, Eiken Chemical Co., Ltd., Tokyo, Japan) on a cobas^®^ c 501 clinical chemistry analyzer (Roche Diagnostics AG, Rotkreuz, Switzerland). On day 0, the cat showed signs of mild hyperbilirubinemia, mild hyperproteinemia, and mild hypoalbuminemia (Table 2 and Figure 2E–G). SAA was low (Table 2 and Figure 2J) and SDMA was in the upper RR (Table 2). On day 4, hyperbilirubinemia was no longer observed (until the end of the observation period) (Figure 2E). Furthermore, the alkaline phosphatase activity was mildly elevated on day 28, and urea concentration was mildly decreased on day 0. Total protein concentration continued to increase (both globulin and albumin concentrations) until day 7. From there on, until the end of therapy, globulin concentration decreased until it was finally within the RR on day 28. Albumin concentration continued to increase until the end of treatment (Figure 2F–H). SDMA concentration was within the RR at all times during treatment. SAA concentration increased to a maximum value on day 14, but was within the RR on day 168 (Figure 2J). All other parameters were within the RR.

### 2.3. Changes in Viral Loads and Anti-FCoV Antibody Titers

**Figure 4:**
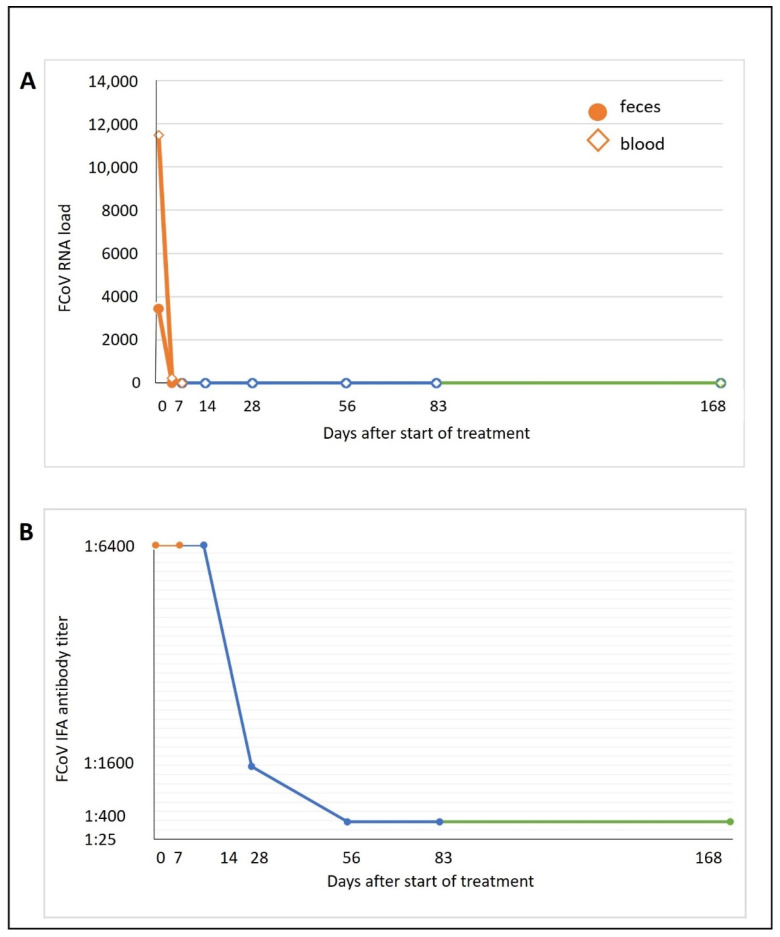
Feline coronavirus (FCoV) viral RNA loads in blood and fecal samples and serum anti-FCoV antibody titer. Lines are colored according to the different study sections: orange, hospitalization in the clinic (days 0–7); blue, recheck visits during treatment (days 14, 28, 56, 83); green, follow-up period at the end of treatment. (**A**) FCoV RNA loads in ethylenediaminetetraacetic acid (EDTA)-anticoagulated blood (presented in copy numbers/mL) and feces (presented in copy numbers/g). FCoV RNA loads were determined by quantitative reverse transcription polymerase chain reaction (RT-qPCR). Dot, feces; rhombus, blood. (**B**). Serum anti-FCoV antibody titer. Antibody titer was determined via indirect immunofluorescence assay (IFA).

The courses of the viral load in blood (on days 0, 4, 7, 14, 28, 56, 83, and 168) and feces (on days 0, 1, 2, 3, 4, 5, 6, 7, 14, 28, 56, 83, and 168) were analyzed by RT-qPCR as previously described [7,8]. Fecal samples were collected using voided samples (on days 0, 2, 3, 4, 5, 6, 7, 14, 28, 56, and 83) or fecal swabs (on days 1 and 168). The viral RNA load in blood before treatment was 11,473 copies/mL blood. On day 4, only 229 FCoV RNA copies/mL blood were detectable. From day 7 onward, no FCoV RNA was detectable in the blood. In feces, excretion of 3437 viral RNA copies/g feces was detectable on day 0. On day 1, only 53 FCoV RNA copies/fecal swab could be detected by RT-qPCR. From day 2, until the end of treatment, viral RNA was no longer detectable in feces (Figure 4A).

Anti-FCoV antibody titers in serum (on days 0, 7, 14, 28, 56, 83, and 168) were determined by indirect immunofluorescence assay (IFA) as previously described [7,8,12,13,14]. The cat exhibited very high anti-FCoV antibody titer levels at the beginning (1:6400 from day 0 until day 14) of the treatment period. From day 56 on, the antibody titer levels decreased to 1:400 (Figure 4B).

### 2.4. Necropsy and Histopathology

A total of 164 days after completion of antiviral treatment, the cat went missing without preceding clinical signs. When the cat was found dead next to the road by the owners, it was immediately submitted to necropsy. A full postmortem examination was performed within 24 h after death. Upon dissection and gross examination, paired samples were taken from all visceral organs and tissues to be (1) snap-frozen for molecular analysis and (2) transferred into 10% neutral-buffered formalin for histopathology and IHC. Fixed samples were trimmed and underwent automated tissue processing, paraffin embedding, and sectioning at 3 µm slice thickness. Sections were routinely stained with hematoxylin–eosin for histopathological evaluation. Further sections of liver and intestine underwent Giemsa and Gram staining.

Upon external inspection and superficial dissection, the carcass had undergone rigor mortis. The body showed prototypic lesions associated with road traffic accidents, including superficial abrasions, subcutaneous and intramuscular hematomas of the head and neck area, and frayed claws. Death occurred due to forced ventral hyperflexion of the head and cervical spine, leading to the luxation of the atlanto-occipital joint and complete spinal cord tear at the medullospinal junction. The other parts of the head, including the OD, were unremarkable. Upon dissection of the body cavities, there were no indications of FIP. In particular, there were no effusions or serosal and subserosal changes. However, generalized lymphadenomegaly of both the internal and peripheral lymph nodes was observed, which was most extensive in the mesenteric lymph nodes, as well as swelling of the tonsils. The spleen only showed mild splenomegaly. Respiratory tract and cardiovascular system, as well as the gastrointestinal tract, liver, and pancreas, were unremarkable. Both kidneys were normal in size; cortex and medulla were clearly delineated and highly unremarkable. No retroperitoneal fluids were evident, and the lower urinary tract was normal.

**Figure 5:**
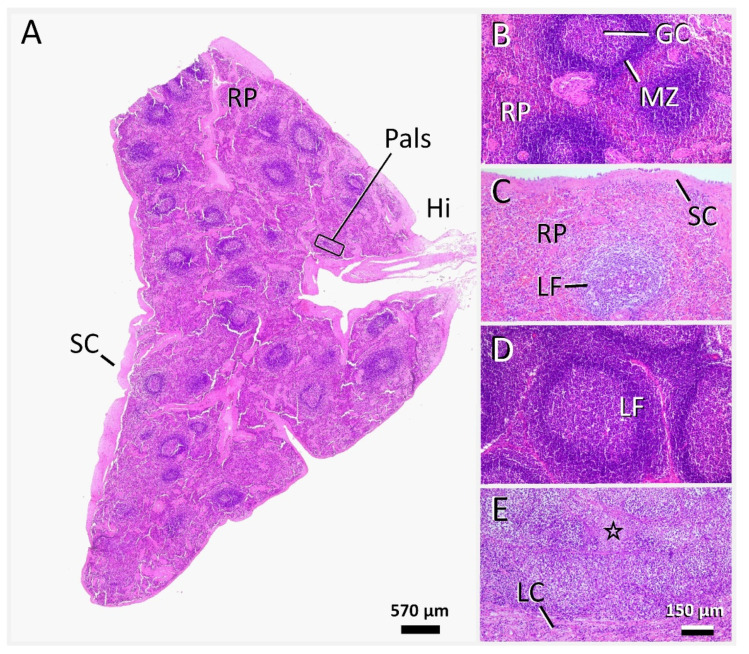
Lymphatic tissue changes in the cat (**A**,**B**,**D**) compared to a cat with active feline infectious peri-tonitis (FIP) (**C**,**E**). Follicular lymphoid hyperplasia was evident in all lymphatic tissues, including the spleen (**A**,**B**), lymph nodes (**D**), and Peyer’s patches and tonsils (not shown). Note the large lymphoid follicles (**A**,**B**; **D**: LF) with prominence of germinal centers (**A**,**D**; **B**: GC) and clearly de-lineated mantle zones (**A**,**D**; **B**: MZ). In the spleen (**A**,**B**), red pulp (**A**,**B**: RP) and periarteriolar lymphoid sheaths (**A**: Pals) were discernible, corresponding to splenic activation type 1 [15]. Le-sions, typically associated with splenic (**C**) and lymph node (**E**) involvement in FIP, including ef-facement of follicular zones and delineation, as well as polymorphonuclear and macrophageal in-filtrate and fibrin deposition (**E**: asterisk), were not seen. Markers: GC, germinal center; Hi, hilus (spleen); LC, lymph node capsule; LF, lymph follicle; MZ, mantle zone; Pals: periarteriolar lym-phoid sheath; RP, red pulp (spleen); SC, splenic capsule. Stain (**A**–**E**): hematoxylin–eosin.

**Figure 6:**
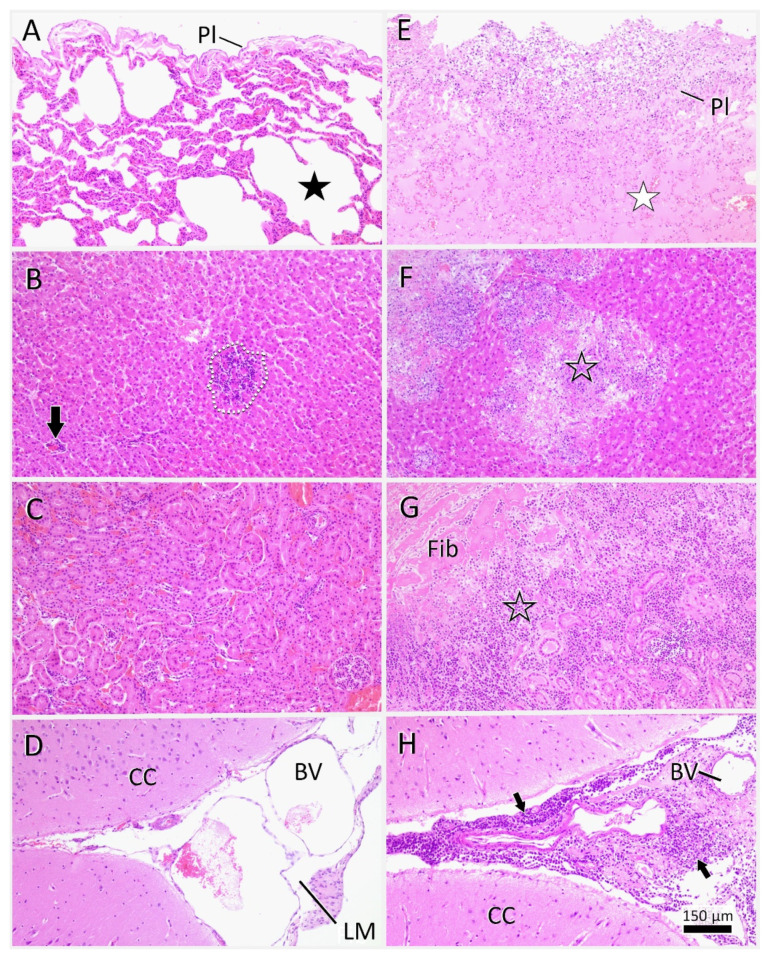
Histology of lungs, liver, kidney, and brain of the cat (**A**–**D**) as opposed to cats with local feline infectious peritonitis (FIP)-associated changes (**E**–**H**). **Lungs**: Note, that the fatally injured cat pre-sented with an alveolar emphysema (**A**: black asterisk), while FIP-typical pleural changes (**E**: Pl) and proteinaceous alveolar oedema (**E**: white asterisk) were not observed. **Liver**: The cat showed further signs of mild lymphocytic periportal hepatitis (**B**: black arrow), and multifocal necrotizing hepatitis (**B**: dashed line). Once again, fibrinoid and pyogranulomatous liver changes in cats with FIP (**F**: empty asterisk) were not evident. **Kidneys**: In the kidneys a mild congestion was seen (**C**). In FIP (**G**), fibrin (**G**: Fib) and pyogranulomatous changes (**G**: empty asterisk) can extend from the capsule into the depth of renal parenchyma with effacement of cortical architecture. **Brain**: Apart from the peracute medullospinal tear, the central nervous system (CNS) condition was unre-markable (**D**), including FIP predilection sites of the leptomeninges (**D**: LM)and brain ventricles (not shown). In CNS manifestation of FIP (**H**), there were mostly ill-defined fibrinonecrotic and pyogranulomatous changes in the meninges intermingling with extensive lymphoid infiltrates (**H**: arrows). Markers: BV, blood vessels; CC, cerebral cortex; LM, leptomeninx; Pl, pleura. Stain (**A**–**H**): hematoxylin–eosin. Scale bar applies to all photomicrographs.

**Figure 7:**
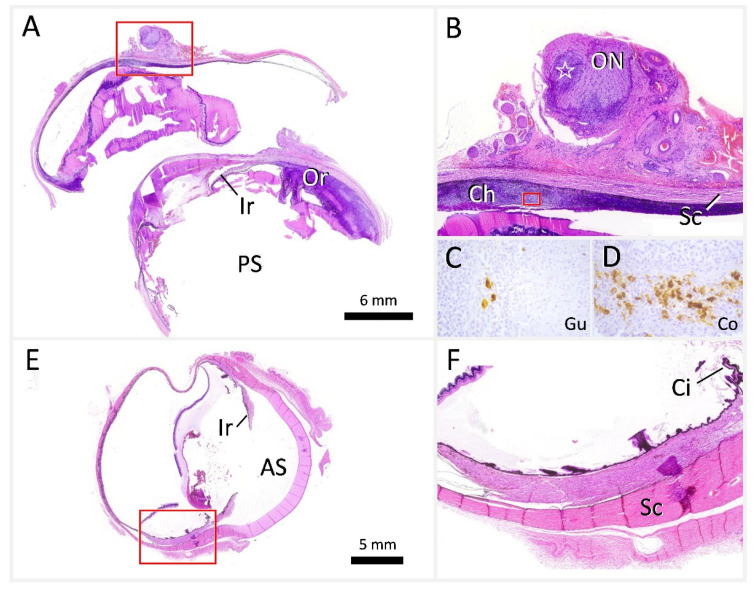
Comparison of the left eye (**A**–**C**), enucleated before antiviral treatment, and the right eye (**E**,**F**), harvested during necropsy. The feline infectious peritonitis (FIP)-affected left eye showed exten-sive pyogranulomatous and lymphocytic inflammation affecting mainly the optic nerve (**A**; **B**: ON, asterisk) and choroid (**A**; **B**: Ch), thereby extending from the posterior pole to the ora serrata (**A**: Or). Fibrin precipitates, proteinaceous fluid, and free-floating cells were abundantly present in both anterior and posterior segments (**A**: PS). On immunohistochemistry of Gusti´s left eye (**C**: Gu), there were scattered brown macrophages immunopositive for feline coronavirus antiges. The signal was similar to that of polymerase chain reaction (PCR)-confirmed control tissue (**D**: Co). No such changes were observed in the right eye (**E**,**F**) from necropsy. Markers: AS, anterior segment; Ch, choroid; Ci, ciliary body; Ir, iris; ON, optic nerve; Or, ora serrata; PS, posterior segment; Sc, sclera. Stain (**A**,**B**,**E**,**F**): hematoxylin–eosin. Stain (**C**,**D**): diaminobenzidine tetrahydrochloride (brown), hematoxylin counterstain. Red frames: localisers of higher magnification pictures: (**A**,**B**; **B**,**C**; **E**,**F**).

Histopathology confirmed the peracute medullospinal injury and excluded pre-existent central nervous system (CNS) changes. The enlargement of the lymphatic tissues histologically corresponded to a severe generalized follicular lymphoid hyperplasia with clearly delineated germinal centers, mantle and marginal zones, and distinct periarteriolar lymphocyte sheaths (Figure 5A,B,D). Histological examination of the other organs also confirmed the absence of FIP-associated changes. The lungs instead presented with a moderate multifocal alveolar emphysema (Figure 6A). Liver sections showed mild oligofocal lymphocytic infiltrates within portal areas and limiting plates, consistent with mild chronic portal and periportal hepatitis. Moreover, there were occasional, randomly distributed and variably sized foci of lytic hepatocellular necroses associated with individual degenerate polymorphonuclear neutrophils and lymphohistiocytic infiltrates (Figure 6B, dashed line). The distribution was typical for the hematogenous spread of bacteria from the guts via the portal vein after passing the mucosal barrier. Accordingly, there were some foci within better-preserved areas of the intestinal mucosa, with an overall increase in the densities of lymphocytes and plasma cells within the propria, accompanied by degenerate polymorphonuclear neutrophils. Hematoxylin–eosin sections, and those stained via Giemsa and Gram staining techniques, exhibited rather diffuse bacterial overgrowths, with mixed morphology ranging from cocci to elongated rods. During necropsy, the OD was removed, and was found to lack any changes indicative of FIP, in contrast to the OS, which had been enucleated prior to antiviral treatment, and showed a fibrotic scar in the area of the ciliary body (Figure 7).

### 2.5. IHC for FCoV Antigen

Tissue sections from all areas sampled (including tonsils, mandibular lymph nodes, mesenteric lymph nodes, spleen, mesenterium, stomach, duodenum, jejunum, cecum, colon, rectum, kidneys, liver, pancreas, brain, spinal cord, OD) underwent immunohistochemical investigation, and tested negative for FCoV antigen expression (Figure 6 and Table 3). IHC was performed using FIPV3-70 monoclonal antibodies (Linaris Biologische Produkte GmbH, Dossenheim, Germany), on formalin-fixed, paraffin-embedded tissue sections as described previously [16]. Negative controls were included in every IHC staining process. These were two brain sections from a cat with confirmed FIP affecting the central nervous system, into which the antibody was substituted using phosphate-buffered saline (PBS), and by an irrelevant mouse monoclonal antibody (Bo-18). Additionally, and to ensure adequate performance of the antibody, a positive tissue control (tissue from a cat with confirmed FIP) was included in every IHC run. Samples were considered positive for FIP in IHC if typical histopathological lesions were present (e.g., pyogranulomatous and fibrinonecrotic tissue lesions at predilection sites with the exclusion of other pathogens), and FCoV antigen was detected within macrophages in these lesions. Samples were considered negative for FIP in IHC if no histopathological lesions suggestive of FIP were detected, and if FCoV antigens were absent in all tissue samples, including lymph nodes (Table 3).

Additional sections from the spleen, lymph nodes, and liver were stained for lymphocyte markers CD3, CD20, and CD79a. In lymphatic tissues, cell phenotypes segregated with the distribution of physiological T cell and B cell areas. Liver infiltrates mainly consisted of T cells, with only a few B lymphocytes.

### 2.6. Tissue FCoV RT-qPCR

From each sampled organ (mandibular lymph nodes, mesenteric lymph nodes, jejunum, duodenum, cecum, colon, rectum, spleen, kidneys, liver, brain, and OD), 30 mg of frozen tissue was transferred to a soft tissue homogenizing tube of the Precellys Lysing Kit CK14 (Labgene Scientific SA, Châtel-St-Denis, Switzerland), and 600 µL of buffer RLT of the RNeasy Mini Kit (Qiagen AG, Hombrechtikon, Switzerland) containing 1% beta-mercaptoethanol (GBiosciences, St. Louis, MO, USA) was added. Tissues were homogenized twice for 1 min at 5000 Hertz on the Precellys 24 tissue homogenizer (Labgene Scientific SA, Châtel-Saint-Denis, Switzerland), followed by a centrifugation at 17,601 x g for 3 min. RNA was extracted from the 600 µL supernatant using the RNeasy Mini Kit (Qiagen) according to the manufacturer’s instructions. RNA was eluted in 30 µL RNase/DNase-free water and stored at −80 °C until further use. All samples were tested undiluted and diluted (1:5) for FCoV RNA by RT-qPCR as described previously [17]. Additionally, all RNA samples from tissues were tested undiluted and diluted (1:5) for the presence of the 18S rRNA housekeeping gene to test for sufficient TNA and absence of potential PCR inhibition. The master mix consisted of 1X Ag-Path RT-PCR buffer (AgPath-IDTM One-step RT-PCR Kit; Applied Biosystems, Rotkreuz, Switzerland), 1.0 μL Array Script reverse transcriptase and AmpliTaq Gold DNA polymerase (AgPath-IDTM One-step RT-PCR Kit; Applied Biosystems), 1X 18s rRNA Dye Mix (VIC/MGB) EUK (Applied Biosystems), and nuclease-free H_2_O was added to a final volume of 20 μL. All FCoVRT-qPCRs were run with 5 μL of TNA in a final volume of 25 μL. Positive and negative controls were run in parallel using a ABI 7500 Fast instrument (Applied Biosystems). An FCoV RNA standard curve was run in parallel as a positive control and to determine the viral RNA copy number [8]. As negative controls, an extraction control (PBS) and DNase/RNase-free water were used. In the 18S rRNA RT-qPCR all tissue tested positive, showing no inhibition at a 1:5 dilution. The lower the cycle threshold (CT) value, the higher the viral load. The cycling conditions were the same as for the FCoV RT-qPCR.

All examined tissue samples, including the OD, were negative for FCoV RNA (Table 3). In comparison, the OS, enucleated before treatment, tested positive for FCoV, with a cycle threshold (CT) value of 25.38.

## 3. Discussion

This case report describes a cat that participated in a clinical trial investigating the efficacy of an oral antiviral drug to treat FIP. Without treatment, virtually all cats suffering from FIP die, making FIP one of the most lethal diagnoses in feline medicine. The survival rate of the 18 cats in this study, however, was 100% [7]. The cat described here was included in the study as it fulfilled the inclusion criteria of (1) a diagnosis of FIP established by IHC, (2) negative test results for FIV and FeLV infection, and (3) absence of other severe diseases. After initial presentation with anterior uveitis, recurrent fever, apathy, and lack of appetite, the cat showed a very swift response to treatment, with rapid improvement of clinical and laboratory parameters leading to full, relapse-free recovery. The cat was treated with 10 mg/kg GS-441524 according to the manufacturer, but it has to be considered that recent additional analysis of the provided drug suggested that a tablet of the multicomponent drug Xraphconn^®^ contains more GS-441524 than officially stated by the manufacturer (data not shown, personal communication J. Horak).

As the analyses show, it is difficult to rely on statements about an unapproved drug. Different illegal antiviral drugs are manufactured under non-standardized conditions. Cats receiving GS-441524 from their owners are treated at variable doses due to the variety of manufactures that produce the drug under uncontrolled conditions. The cats in the cited study were treated with a significantly higher dose than assumed, and only mild side effects occurred [7]. Whether the cat would have been virus-free with a lower dose requires further research. 

The main clinical abnormality of FIP in the cat presented in this case report was anterior uveitis, manifesting as anisocoria, aqueous flare, and an optic neuritis with retinal detachment, which is consistent with the typical clinical signs observed in cats with ocular FIP [18]. FIP is the most commonly identified cause of uveitis in young cats. According to a study of the North Carolina Veterinary School, 19 of 120 cats (15.8%) with uveitis had FIP [19]. A study from the UK showed a similar distribution pattern, with FIP being the cause of uveitis in 15/92 cats (16.3%) [18]. Confirmation of FIP as the cause of uveitis is often difficult. Clinical signs are not pathognomonic, and aqueous humor analysis, including cytology and screening for infectious diseases is often unspecific [20,21,22]. Detection of FCoV antigens within macrophages via immunostaining of ocular tissue can confirm the diagnosis, although false-positive results are possible using immunocytochemistry on aqueous humor [23]. In the cat of the present case report, the OS was enucleated to confirm FIP. Histopathology of the OS revealed marked pyogranulomatous uveitis, pyogranulomatous neuritis of the optic nerve, and retinal detachment. In IHC, multiple macrophages were positive for FCoV antigen. In contrast, the OD had shown no abnormalities on ophthalmic examinations, although at necropsy, a fibrotic scar could be seen on the OD in the area of the ciliary body. It is conceivable that the virus infiltrated not only the OS, but also the contralateral eye, and that these “microlesions” were too minor to be clinically conspicuous and recognized by ophthalmic examination. In both IHC for FCoV antigen and tissue-based FCoV detection using RT-qPCR, FIP could no longer be detected in the enucleated eye postmortem, suggesting cure by treatment with Xraphconn^®^. Thus, the scarring could be an indication of previous lesions caused by FIP after healing.

A case report from the US described treatment of four cats with FIP and neurological and/or ocular signs [24]. The cats were treated with GS-441524 via subcutaneous injections at a dose of 5–10 mg/kg, applied once daily for at least 12 weeks. All of the four cats responded to treatment initially, including remission of ocular signs. Serial ophthalmic examinations revealed healing of ocular changes presenting as chorioretinal scars, similar to the changes in the cat described in the present case report. However, one cat of the case series of [24] experienced two relapses, and was ultimately euthanized after two courses of treatment. Postmortem examination revealed lymphocytic, histiocytic uveitis, and choroiditis, and viral antigens could be detected in various tissues, including the eye, by IHC. This could either be caused by viral persistence or recurrent FCoV infection and mutation. This cat received GS-441524 at a lower dose (5 mg/kg) than the cat described in this current case report. It is known that drug levels of GS-441524 in aqueous humor are lower than in serum [5]; thus, it is likely that the higher dose used in the present study was more effective [24]. This was corroborated by the fact that results from both IHC and RT-qPCR conducted on ocular and other tissues were negative for FCoV antigen and RNA, respectively, in the cat in the present report. Further studies are needed to investigate whether an intermediate dose of GS-441524 might be adequate to permanently stop viral replication in cats with ocular FIP.

The medullary rim signs, which was visible in both kidneys, also disappeared towards the end of treatment. Medullary rim signs can have various causes, but has been described in association with FIP. In a retrospective study including 243 cats showing medullary rim signs, 15 of these cats were finally diagnosed with FIP [25]. Therefore, treatment with GS-441524 likely also cured FIP-associated changes in the cat’s kidneys.

No residual FIP lesions were present in the cat 164 days after the end of treatment with the multicomponent drug Xraphconn^®^, apart from a generalized lymphadenopathy due to massive lymphoid hyperplasia. The involvement of mesenteric lymph nodes could partially resemble a consequence to the presumed mild suppurative bacterial enteritis. Gastrointestinal infection and the subsequent portogenic involvement of the liver, on the other hand, cannot explain lymphoid hyperplasia at distant sites such as the tonsils, and mandibular and superficial cervical lymph nodes. The presence of lymphadenopathy could be an incidental unrelated finding, possibly caused by a recent (re)infection with FCoV, or could be an indication of a “long FIP syndrome”. It is possible that some cats have a genetic predisposition of developing FIP. According to current theory on FIP pathogenesis, FIP occurs within cats that are genetically predisposed to being unable to control viral replication effectively, resulting in uncontrolled virus replication and increased opportunity for mutations. These mutations lead to a switch in pathogenicity of the virus, resulting in a variant that is able to efficiently replicate in macrophages. It is the ability to replicate in macrophages in an uncontrolled fashion that distinguishes FIP-causing variants from low-pathogenic feline coronavirus isolates [26,27]. To further characterize the predisposition to developing FIP, single-nucleotide polymorphisms (SNP) in the feline IFN-γ gene were previously investigated, and certain genotypes were described as FIP-susceptible factors [28,29]. It was also demonstrated that cats with FIP have lower IFN-γ production than cats infected with FCoV without FIP [30,31,32,33]. In the present cat, there were other known cases of FIP in the cat’s family history, which suggests a genetic predisposition of this family. However, FCoV infection neither as relapse nor as newly acquired was found by IHC or RT-PCR that would explain the lymphadenopathy. Lymphadenopathy could be considered as a sign of an exaggerated genetically conditioned reaction of the immune system. Also, persistence of lymphadenopathy after elimination of the FCoV could be discussed as a “long FIP syndrome”. In human medicine, cases have been described in which viral RNA persisted after clinical resolution of an acute infection, such as Severe Acute Respiratory Syndrome Coronavirus 2 (SARS-CoV-2), the long COVID syndrome [34]; however, what is detected by RT-PCR is mostly fragmented RNA strains [35]. In the present case report, RT-PCR did not test positive in any tissue of the cats, and thus, no fragmented persisting RNA was found. Nonetheless, lymphadenopathy might be an aftereffect of FIP, without the presence of virus. Further studies are needed to prove whether these changes are due to a long-term adverse effect of treatment or potentially associated with a “long FIP syndrome”. 

The initially seen fluid in the retroperitoneal space was not present in the postmortem examination. In FIP, lesions are induced by immune complexes deposited at the wall of blood vessels, subsequently activating the complement cascade, and damaging vascular tissues [36]. In addition to the spleen, the omentum and the mesenteric lymph nodes are tissues with high viral loads [37]. However, no virus was presented in the lymphoid tissue of the cat analyzed here. Moreover, it remains to be determined whether the preceding FIP might have paved the way for intestinal infection e.g., by affecting local or systemic immunocompetence.

FCoV was no longer detected in any tissue of the cat. In addition, viral load decreased in the blood within a short period of time after treatment initiation, and fecal shedding stopped by day 2. These results indicate that the cat was 100% cured of FIP. The antibody titer decreased during treatment, although it did not become negative. Possibly, the titer would have continued to decrease after infection was cleared, as described previously in FCoV-infected cats without FIP. Anti-FCoV antibodies can sometimes be measured after the clearance of (harmless) FCoV infection for several months, and this is not a sign of viral persistence [38].

FIP relapses after treatment with GS-441524 have been described in a few cases [5]. In these cases, the question arises whether a reinfection with FCoV and a new mutation took place, or whether the virus could not be completely eliminated in these cats and was still present in individual tissues. The presently described cat was 100% free of the virus, making relapses after appropriate treatment very unlikely. Unfortunately, the drug used for the treatment of FIP in this cat is currently not legally available for veterinary use in most countries, forcing well-meaning owners to self-diagnose and treat their cats based on judgment of non-veterinary lay people and social media groups. Thus, there is an urgent need for respective official bodies and industry to work towards a swift licensing process of the drug, so that it can be legally used by veterinary experts to offer supervised treatment to cats suffering from FIP. The limitation of this case report is that only one cat was examined via necropsy. The other study cats are still alive at the time of publication.

## 4. Conclusions

This is the first report describing clinical and laboratory as well as postmortem findings in a cat cured of FIP that subsequently died accidentally. GS-441524 was highly effective in this cat, and neither signs of FIP nor FCoV RNA or antigens could be detected postmortem. GS-441524 is currently the most effective treatment of FIP, and should be licensed for veterinary use as soon as possible.

## Figures and Tables

**Table 1:** Karnofsky’s score adapted from Hartmann and Kuffer [10,11].

0%	Dead
20%	severely diseased
40%	major changes in the general condition
60%	medium changes in the general condition
80%	minor changes in the general condition
100%	completely normal general condition

**Table 2:** Physical examination and laboratory parameters of a 6-month-old cat with feline infectious peritonitis. Columns are colored according to the different study sections: orange, hospitalization in the clinic (day 0–7); blue, recheck visits during treatment (day 14, 28, 56, 83); green, follow-up period after the end of treatment. Values marked in bold are outside the reference ranges.

		Day 0	Day 1	Day 2	Day 3	Day 4	Day 5	Day 6	Day 7	Day 14	Day 28	Day 56	Day 83	Day 168
**Clinical parameters**														
**Effusion ^1^**		+/–	–	–	–	–	–	–	–	–	–	–	–	–
**Body weight (kg)**		1.81	1.90	1.93	1.94	1.93	1.98	2.00	2.06	2.21	2.47	3.02	3.20	3.89
**Karnofsky score (%)**		70	80	80	95	95	95	100	100	100	100	100	100	100
**Appetite ^2^**		+	+	++	++	++	+++	+++	+++	++++	++++	++++	++++	++++
**Temperature (°C)**		**40.5**	38.5	38.7	38.1	37.8	37.8	38.4	38	38.3	37.7	38.4	38.9	38.3
**Laboratory parameters**														
**Hematocrit (L/L)**		**0.176**		**0.207**		**0.188**			**0.252**	**0.289**	**0.328**	0.382	0.334	0.479
**Mean corpuscular volume (fL)**		**38.0**		**39.3**		**39.8**			43.8	43.6	40.8	43.0	**39.3**	51.6
**Mean corpuscular hemoglobin (fmol/L)**		**0.799**		0.816		0.805			0.852	0.905	0.872	0.866	0.872	**1.250**
**Mean corpuscular hemoglobin concentration (mmol/L)**		21.0		20.8		20.2			19.4	20.8	21.3	20.2	**22.2**	**24.2**
**Reticulocytes (10^9^/L)**		18.1		50.1		133.6			78.2	20.6	20.9	31.1	41.6	115.1
**WBC ^3^ (10^9^/L)**		10.49		**13.61**		**19.03**			**12.41**	9.33	**13.10**	11.00	**11.38**	**5.39**
**Granulocytes (10^9^/L)**	**Neutrophils**	8.71		7.28		10.78			6.87	5.54	5.97	4.71	3.56	3.57
	**Eosinophils**	0.01		0.16		0.28			0.24	0.50	0.47	0.37	0.59	0.03
	**Basophils**	0		0.01		0.01			0.01	0.01	0.01	0.01	0.01	0.01
**Monocytes (10^9^/L)**		**0.64**		**0.91**		**0.93**			0.47	0.38	0.23	0.14	0.26	0.22
**Lymphocytes (10^9^/L)**		1.13		**5.25**		**7.03**			**4.82**	2.90	**6.42**	**5.77**	**6.96**	1.56
**Thrombocytes (10^9^/L)**		**101**		277		452			519	265	345	396	478	205
**Liver enzyme activity (IU/L)**	**ALT ^4^**	36		Np ^5^		44			40	30	42	60	61	54
	**AP ^6^**	14		np		24			37	63	**106**	80	77	56
**Urea (mmol/L)**		**4.5**		np		5.0			5.0	6.7	7.4	7.6	6.5	8.5
**Creatinine (µmol/L)**		62		np		53			50	59	81	88	110	107
**Symmetric dimethylarginine (µg/dL)**		17.0		13.0		14.0			14.0	11.0	12.0	15.0	14.0	10.0
**Bilirubin (µmol/L)**		**5.2**		np		2.1			2.5	1.5	0.6	0.8	1.1	0.4
**Total protein (g/L)**		**109.1**		np		**111.2**			**109.4**	91.5	78.6	67.7	71.0	71.4
**Albumin (g/L)**		**25.1**		np		26.9			26.4	27.6	34.1	37.5	40.6	42.8
**Globulin (g/L)**		**84.0**		np		**84.3**			**83.0**	**63.9**	44.5	30.2	30.4	28.6
**Albumin/globulin ratio**		**0.30**		np		**0.32**			**0.32**	**0.43**	0.77	1.24	1.34	1.50
**Serum amyloid A (mg/L)**		0.5		**6.3**		**5.3**			**4.2**	**19.6**	**5.8**	1.0	**5.1**	0
**Viral loads**														
**FCoV ^7^ RNA feces (copies/g)**		3437	53	0	0	0	0	0	0	0	0	0	0	0
**FCoV RNA blood (copies/mL)**		11,473				229			0	0	0	0	0	0
**Antibody titers**														
**FCoV antibody titer (IFA ^8^)**		1:6400							1:6400	1:6400	1:600	1:400	1:400	1:400

^1^ Effusion was graded from +++ (high-grade) to–(no effusion); ^2^ Appetite was graded from + (reduced appetite) to ++++ (normal appetite); ^3^ WBC, white blood cells; ^4^ ALT, alanine aminotransferase; ^5^ np, not performed; ^6^ AP, alkaline phosphatase; ^7^ FCoV, feline coronavirus; ^8^ IFA, indirect immunofluorescence assay.

**Table 3:** Results of immunohistochemistry (IHC) for feline coronavirus (FCoV) antigen and quantitative reverse transcriptase PCR (RT-qPCR) in different tissues compared to control tissues.

Tissue	IHC for FCoV Antigen	FCoV RT-qPCR ^1^ (Viral Load)	18S rRNA RT-qPCR ^1^ (RNA Quality Control) CT ^2^-Value
mandibular lymph node	negative	negative	15.09
jejunum	negative	negative	15.86
duodenum	negative	negative	14.08
spleen	negative	negative	14.18
colon	negative	negative	16.02
mesenterial lymph node	negative	negative	15.57
kidney	negative	negative	20.62
caecum	negative	negative	14.28
rectum	negative	negative	15.21
liver	negative	negative	20.54
brain	negative	negative	20.82

^1^ RNA diluted 1:5; ^2^ cycle threshold values.

## Data Availability

The authors confirm that the datasets analyzed during the study are available from the corresponding author upon reasonable request.
